# Extracts from *Chlorella vulgaris* Protect Mesenchymal Stromal Cells from Oxidative Stress Induced by Hydrogen Peroxide

**DOI:** 10.3390/plants12020361

**Published:** 2023-01-12

**Authors:** Maria G. Savvidou, Ioulia Georgiopoulou, Nasia Antoniou, Soultana Tzima, Maria Kontou, Vasiliki Louli, Chronis Fatouros, Kostis Magoulas, Fragiskos N. Kolisis

**Affiliations:** 1 Biotechnology Laboratory, School of Chemical Engineering, National Technical University of Athens, Iroon Polytechniou Str., Zografou Campus, 15780 Athens, Greece; 2 Laboratory of Thermodynamics and Transport Phenomena, School of Chemical Engineering, National Technical University of Athens, 9 Iroon Polytechniou Str., Zografou Campus, 15780 Athens, Greece; 3 TheraCell Advanced Biotechnologies, 14564 Kifisia, Greece

**Keywords:** microalgae, oxidative stress, bioactive components, *Chlorella vulgaris*, mesenchymal cells, supercritical fluid extraction, antioxidant activity, cell viability

## Abstract

Microalgae as unicellular eukaryotic organisms demonstrate several advantages for biotechnological and biological applications. Natural derived microalgae products demand has increased in food, cosmetic and nutraceutical applications lately. The natural antioxidants have been used for attenuation of mitochondrial cell damage caused by oxidative stress. This study evaluates the in vitro protective effect of *Chlorella vulgaris* bioactive extracts against oxidative stress in human mesenchymal stromal/stem cells (MSCs). The classical solid-liquid and the supercritical extraction, using biomass of commercially available and laboratory cultivated *C. vulgaris*, are employed. Oxidative stress induced by 300 μM H_2_O_2_ reduces cell viability of MSCs. The addition of *C. vulgaris* extracts, with increased protein content compared to carbohydrates, to H_2_O_2_ treated MSCs counteracted the oxidative stress, reducing reactive oxygen species levels without affecting MSC proliferation. The supercritical extraction was the most efficient extraction method for carotenoids resulting in enhanced antioxidant activity. Pre-treatment of MSCs with *C. vulgaris* extracts mitigates the oxidative damage ensued by H_2_O_2._ Initial proteomic analysis of secretome from licensed (TNFα-activated) MSCs treated with algal extracts reveals a signature of differentially regulated proteins that fall into clinically relevant pathways such as inflammatory signaling. The enhanced antioxidative and possibly anti-inflammatory capacity could be explored in the context of future cell therapies.

## 1. Introduction

Algal extracts have been investigated for their efficacy in preventing a variety of human health disorders. Metabolically derived microalgae compounds have been studied for their possible clinical and medical applications [1,2]. The synthesis of these products can be metabolically regulated via micro- macro- nutrients [3,4] and can be enhanced via magnetic immobilization [5,6,7] while their extraction has recently been improved by magnetic harvesting techniques [8,9]. Microalgae produce bioactive compounds with pharmaceutical and therapeutic applications such as antibiotics, hepatotoxic and neurotoxic compounds, and enzymes. These microalgae-produced compounds cannot be synthesized easily, affordably and rapidly by chemical methods, thus their direct extraction from cells is advantageous. The benefit of these compounds compared to other sources is their increased production efficiency followed by reduced production time. Microalgae pigments, such as chlorophylls and carotenoids, purportedly have also shown positive impacts on human health, such as in providing rapid cell growth and repair, and preventing cancer, cardiac diseases, neurological disorders, and eye diseases [10,11,12]. *Chlamydomonas reinhardtii* is the most extensive used microalgae in pharmaceutical biotechnology; however, *Chlorella*, *Dunaliella*, and *Scenedesmus* species produce metabolically derived compounds with important clinical applications for human health [13] and promising results. Moreover, the microalgae-derived products, such as carotenoids, have been found to have either a direct action or to regulate the expression of genes involved in antioxidant pathways [14]. The orientation of the microalgae cell cultures can support the above referred advantages as well as high biodiversity and metabolic plasticity [15].

*C. vulgaris* is a microalga used for animal feed, food supplement, aquaculture, cosmetics and is presumed to have untapped potential for the pharmaceutical industry [16]. It can easily be cultured and harvested, with short generation times allowing for an environmentally friendly drug discovery approach [17]. However, only a few studies have been performed about the phytochemical, antioxidant, and pharmacological activities of this microalgae [18]. Various microalgae derived compounds such as ascorbic acid, glutathione, tocopherols, carotenoids and chlorophyll demonstrate antioxidant properties [15]. Phenolic compounds and acetone extracts from *Chlorella* species have been studied for their anti-oxidant activity, as well as aqueous extracts of various microalgae species [15,19,20]. Likewise, *C. vulgaris* crude extracts obtained under various stress conditions have been evaluated for their antioxidant activities [18]. Furthermore, polysaccharides isolated from *C. vulgaris* increased the lifespan of *Caenorhabditis elegans* under induced oxidative stress [21]. 

It is known that oxidative stress can cause cellular damage resulting in inflammation and misregulated metabolism, which are common culprits in many pathological situations. Reactive Oxygen Species (ROS) have been long assumed to be damaging to cell function and have been shown to cause apoptosis and senescence of mesenchymal stromal/stem cells (MSCs) in vitro [22]. ROS are short-lived oxygen-containing molecules with chemical reactivity towards nucleic acids, proteins, and lipids. Human cells, including MSCs, are able to neutralize ROS by the mitochondrial antioxidant enzyme, superoxide dismutase 2 (SOD2); however, elevated levels of ROS, defined as oxidative stress, arrest the MSC cell cycle and can trigger apoptosis [23]. MSCs, due to their self-renewal, are widely used in cell-based regenerative medicine. They have been under investigation for treating diverse diseases, with promising outcomes achieved in animal models and clinical trials. Their mechanism of action is multi-faceted and is based on secreted peptide and ligands and related with tissue regeneration, trophic/anti-inflammatory secretion, and immunomodulation [24]. They act to promote regeneration of injured/diseased tissues after transplantation in vivo, contributing to tissue engineering. Increased ROS encountered by MSCs in their microenvironment is an extra obstacle in their efforts to re-establish homeostasis and suppress inflammation. Supplementing MSCs with algal extracts that can exert antioxidant function may have the potential to enhance their therapeutic activity.

Here, we attempted to evaluate the in vitro protective effect of extracted bioactive components from microalgae *C. vulgaris* against oxidative stress in human MSCs by comparing the antioxidant potential of algal biomass obtained by different extraction methods. To this end, the conventional solid-liquid extraction using aq. ethanol as solvent and the novel supercritical fluid extraction with carbon dioxide (CO_2_) were employed.

## 2. Results

### 2.1. Biomass Profile of the Commercially Available and Laboratory Cultivated C. vulgaris

The primary composition of the commercially available biomass is described in previous work [25] and is presented in Table 1, along with the proximate analysis of the laboratory cultivated *C. vulgaris*. The establishment of the biomass profile supports the quality of either the commercially available or laboratory cultured *C. vulgaris* biomass and its antioxidant activity due to the produced constituents. 

### 2.2. Bioactive Compound Recovery and Antioxidant Activity Measurement of the C. vulgaris Extracts

Individual extracts of *C. vulgaris* from commercially available biomass via supercritical extraction with CO_2_ (SFE com.), solid-liquid extraction with 90% ethanol (SLE com), and from laboratory cultivated biomass via solid-liquid extraction with 90% ethanol (SLE cult) are collected. These three extracts are subsequently compared in their antioxidant capacity to determine whether the different extraction approaches or the microalgae source (cultured or not *C. vulgaris*) influence the anti-oxidative attributes. The results of extract characterization are presented in Table 2.

**Figure 1:**
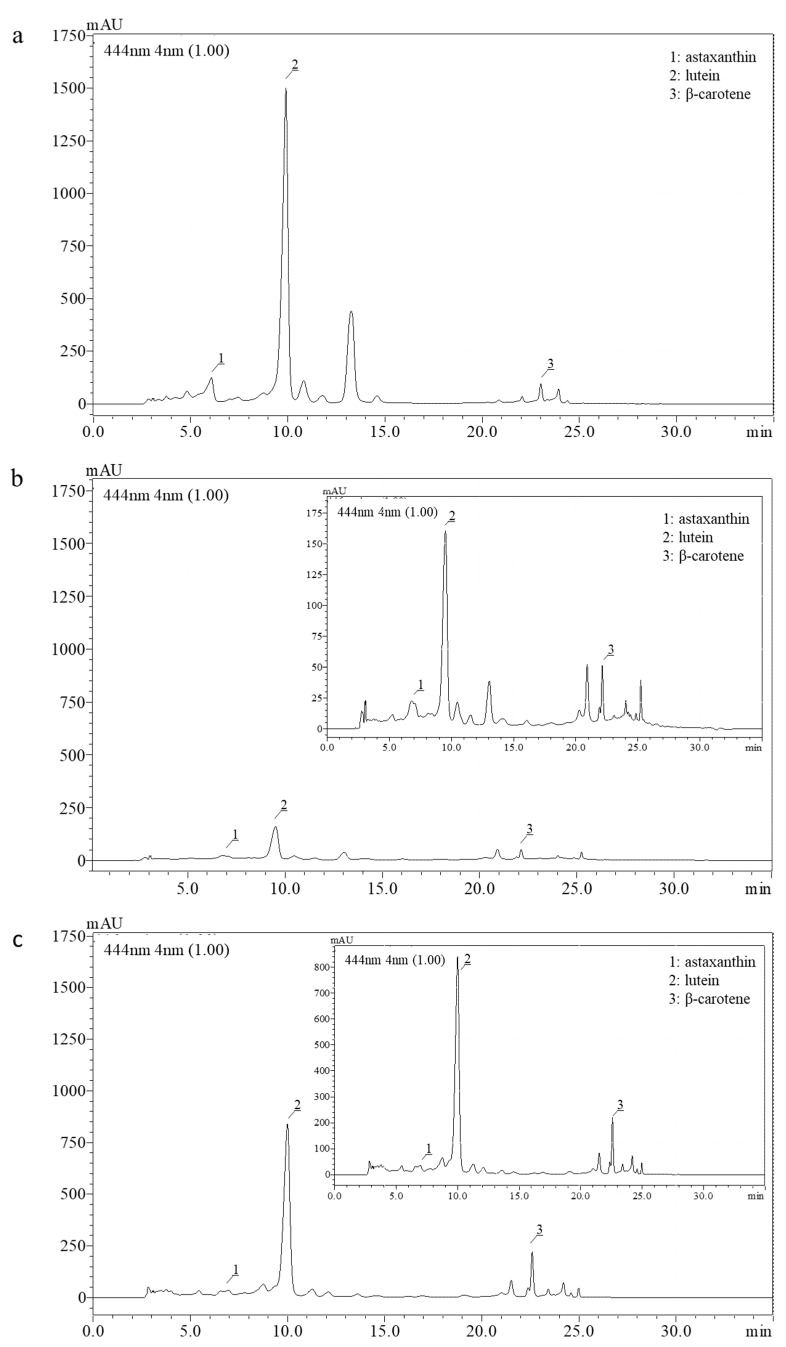
HPLC chromatograms of (**a**) SLE cult (concentration 7.3 mg/mL); (**b**) SLE com. (concentration 5.2 mg/mL) and (**c**) SFE com. (concentration 7.1 mg/mL) extracts at 444 nm. (**b**,**c**) include magnification of the chromatograms (**upper right**) for better viewing. The identified carotenoids are numbered and listed.

Although significantly higher yield extract is recovered during SLE com. compared to SFE com., the SFE com. extract presented more than double carotenoid content. The chromatograms obtained from the RP-HPLC analysis as well as the retention time of the identified carotenoids are presented in Figure 1 and Table 3, respectively. Out of the well-separated peaks of all three profiles, the carotenoids of our interest were identified based on their retention time (Table 3) and the absorbance spectra of the corresponding external standards (Appendix A).

Regarding the rest bioactive compounds, the two methods and solvent choice did not seem to affect the amount of phenols; however, chlorophyll content is enhanced during SLE com. SLE cult. presented comparable yield with SLE com., and its extract was richer in carotenoids and total chlorophyll content compared to SLE com. or SFE com. However, the total phenolic content is lower resulting in a weaker antioxidant activity as measured by a DPPH free radical scavenging assay (Table 2). In conclusion, the antioxidant activity of the commercially available obtained *C. vulgaris* extract is enhanced compared to the laboratory cultured one, while the extract derived from the supercritical extraction method demonstrated increased antioxidant efficiency compared to the conventional SLE method.

### 2.3. Effect of C. vulgaris Extracts Obtained by Different Extraction Methods on Cell Viability

**Figure 2:**
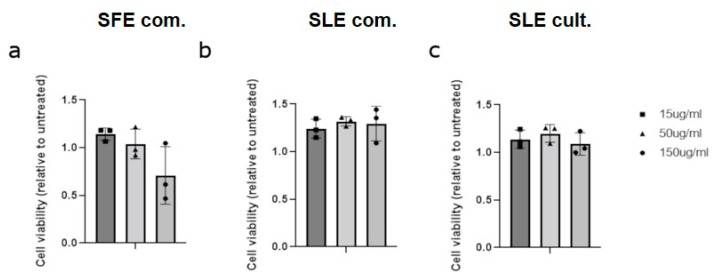
Effect of *C. vulgaris* extracts obtained by different extraction methods on cell viability. Quantification of cell viability of human mesenchymal stem cells (MSCs) treated with *C. vulgaris* (**a**) SFE; (**b**) maceration commercially available and (**c**) maceration laboratory cultivated for 24 h examined by cell viability assay. Data represent mean ± SEM (Comparisons by ANOVA with Tukey’s multiple comparisons test, *n* = 3 independent experiments).

In order to study the antioxidant efficiency of the microalgae extracts on ROS-induced MSCs, any possible cytotoxic effects derived from these extracts to mesenchymal stem cells are examined via cell viability assay. Proliferation of human mesenchymal stem cells is not significantly decreased after 24 h, when exposed to 150 μg/mL of extract derived from the SFE com. method (Figure 2a). There was no significant change in cell proliferation with any other extract dilution (Figure 2b,c).

### 2.4. C. vulgaris Extracts Mediate the Inhibition of H_2_O_2_-Induced ROS Generation in MSCs

**Figure 3:**
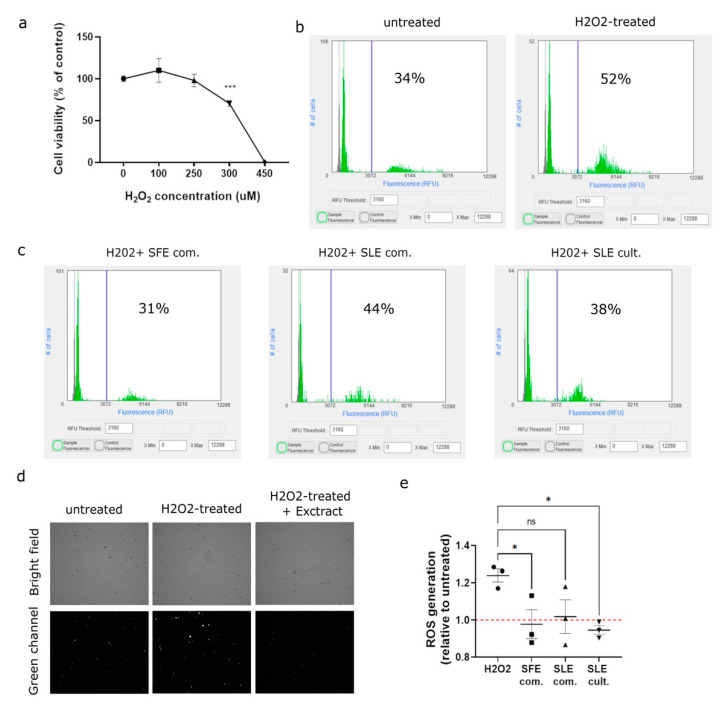
*C. vulgaris* extracts mediate the inhibition of H_2_O_2_-induced ROS generation in MSCs. (**a**) Killing curve of MSCs treated with increasing concentrations of H_2_O_2_ for 24 h. Data represent mean ± SEM (Comparisons by ANOVA with Dunnett’s multiple comparisons test, *** *p* < 0.001, *n* = 3 independent experiments); (**b**) representative histograms of DCF-DA fluorescence of H_2_O_2_-treated cells and control untreated cells. Numbers in the histograms represent the percentage (%) of cells with fluorescence intensity above the threshold as determined by the background noise from unstained cells, scoring positive for green signal; (**c**) representative histograms of DCF-DA fluorescence of H_2_O_2_-treated MSCs when pre-incubated with *C. vulgaris* extracts. Numbers in the histograms represent the percentage (%) of cells scoring positive for green signal (similarly as in (**b**)). (**d**) representative bright field and green fluorescence images of MSCs recorded from the automated fluorescent cell counter from three different conditions: untreated cells (**left**), H_2_O_2_-treated (**middle**), extract+H_2_O_2_-treated (**right**); (**e**) quantification of reactive oxygen species (ROS) generation in H_2_O_2_-treated MSCs when pre-incubated with *C. vulgaris* extracts. Quantification of fluorescence intensity was performed relative to untreated cells. Data represent mean ± SEM (Comparisons by ANOVA with Dunnett’s multiple comparisons test, * *p* < 0.05, *n* = 3 independent experiments, ns = non-significant).

The efficiency of microalgae extracts rescuing from the oxidization phenotypes in MSCs is identified. Treatment with H_2_O_2_ for 24 h reduces the cell viability in a concentration-dependent manner, and this is used as a proxy for the amount of oxidative damage in MSCs. As shown in Figure 3a, 300 μM H_2_O_2_ significantly decreased the cell viability of MSCs compared to the control (70.4 ± 3.5%, *p* < 0.001). This concentration is used in the subsequent experiments since it is verified that normal cell function is compromised, and it lies in the middle of the dose–response curve. Higher H_2_O_2_ concentrations led to increased cell death/irreversible oxidative damage that do not allow a successful comparison with this assay. To determine the involvement of reactive oxygen species (ROS) in apoptosis, the DCF-DA assay is used and changes in the intracellular ROS levels are measured, since DCF-DA becomes proportionally more intensely green fluorescent when oxidized by free radicals. Exposure of MSCs in exogenous H_2_O_2_ 300 μM is associated with increased intracellular ROS generation (Figure 3b,d). Next, we investigated the impact of *C. vulgaris* extracts on ROS generation in H_2_O_2_-treated MSCs. Interestingly, pretreatment of MSCs for 24 h with microalgae extracts (at 50 µg/mL) significantly attenuated the observed increase in ROS levels, as shown in Figure 3c–e.

### 2.5. Proteomic Analysis of MSC Secretomes after Pre-Incubation with Algal Extracts

**Figure 4:**
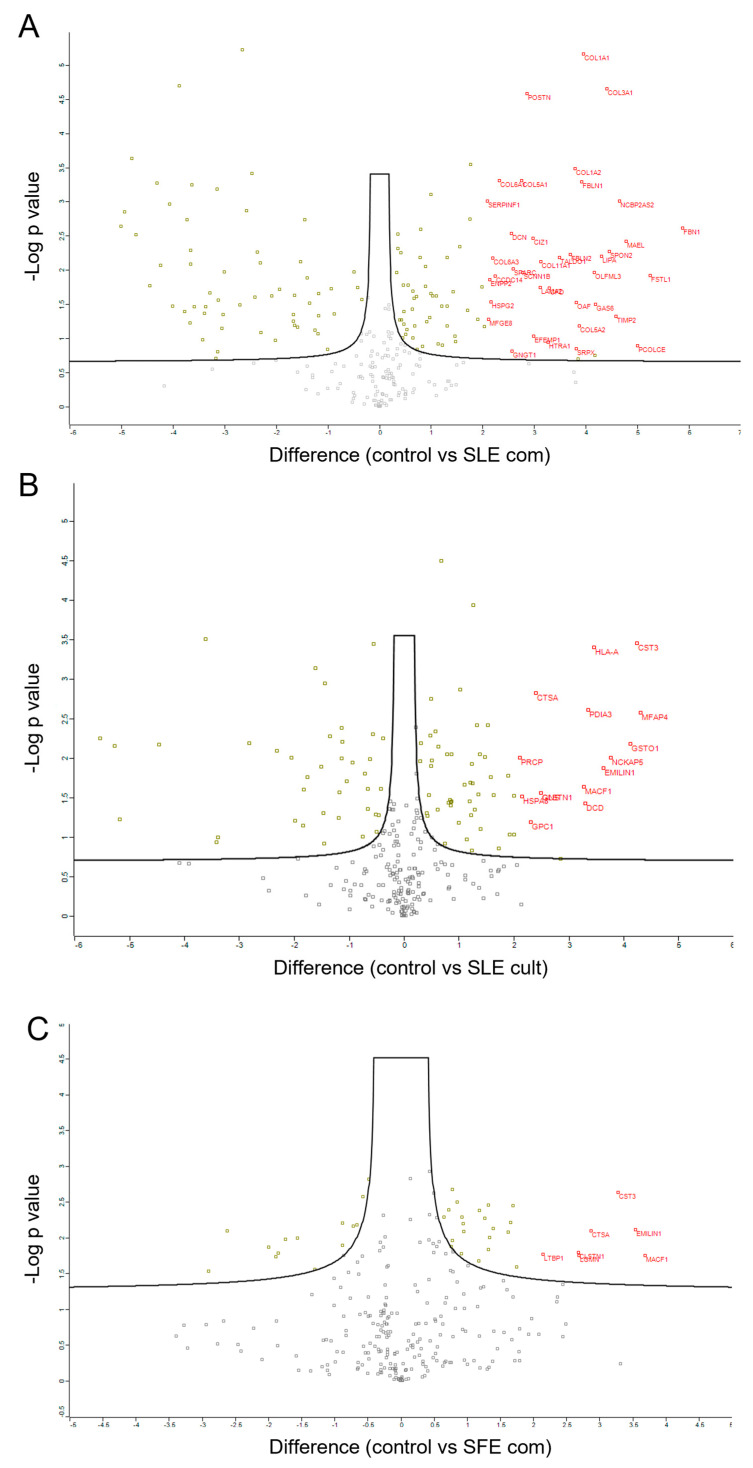
Volcano plots of differentially regulated proteins detected by LC-MS/MS Mass Spectrometry. *C. vulgaris* extracts mediate changes in the paracrine function of licensed MSCs, detected as differentially regulated proteins in their secretome. The *y*-axis is the -Log of the *p*-value while the *x*-axis is the fold difference relative to reference control, with 0 (no difference) being in the middle of the volcano plot. The black curve denotes the statistical significance limit of α = 0.05 (*p*-value has to be less than 0.05 for significance). Dots that fall below the black curve do not pass the significance test (light gray dots). Dots on the left of the curves are downregulated proteins, dots on the right correspond to upregulated proteins. Significantly upregulated proteins of more than 2-fold are tagged with their name in red next to the corresponding dot. (**A**) marks the secretome signature of licensed MSCs that were pre-treated with SLE com; (**B**) is for SLE cult and (**C**) for the SFE com. All three samples were compared to a reference control that did not receive extracts. Gray square dots in the volcano plot mark the detected proteins that were not significantly differentially regulated in the secretome. Gold-yellow squares mark the proteins that were significantly up- or downregulated in each dataset (upregulated scatter to the right of the volcano plot and downregulated to the left). With red letters, the names of the upregulated proteins are marked next to each dot. For better visibility please refer to Supplementary Table S1 or the digital version of this figure.

To gain insights into the functional consequences of the extracts acting on the MSCs, a proteomics approach was undertaken. A major mode of action of the MSCs in a cell therapy setting is their secretory behavior, also known as paracrine function, which encompasses the secretion of various proteins, growth factors, and extracellular vesicles [27]. As described in Methods Section 4.3.8, the cells were pre-treated with the extracts and, after washout, they were stimulated (licensed MSCs) and left to produce their inflammatory response conditioned secretome. As control, licensed cells that had not received extracts were used. This was performed in order to find differentially regulated proteins in the inflammatory secretome signature, as a consequence of incubation with extracts, and not in response to inflammatory stimulus, as all samples were stimulated with TNFα and were thus licensed. The collected secretomes were analyzed by LC-MS/MS using the DIA method. As seen in Figure 4, the pre-incubation with extracts led to a significant differential regulation of a subset of secreted factors, especially for the SLE extracts, denoting a palpable effect in the paracrine function of the MSCs. The SFE extract also caused changes, but to a lesser extent (an order of magnitude less differentially regulated proteins). In the volcano plots of Figure 4, the upregulated proteins are marked with their names in red; as an example, downregulated proteins are not tagged for simplicity. Details on all hits detected by this proteomics approach are tabulated in Supplementary Table S1 accompanying this paper, as output from the Perseus software. In this Excel table, there is a separate sheet for each comparison (SLE_com vs. control secretome, SFE_com vs. control, SLE_cult vs. control) plus a 4th sheet with the cumulative comparisons of expression levels for each protein detected in the secretome of licensed MSCs (see Supplementary Table S1). In this table, the statistical significance is marked by a “+” sign in the respective column, the “Difference” column marks the fold-difference in expression levels (positive for upregulated while negative for downregulated, always compared to control) and the numeric values in the sample columns correspond to the logarithmic intensities for each protein as detected by the MaxQuant Software used to process the raw LC-MS/MS Orbitrap data. In particular, this dataset reveals that, when MSCs get stimulated in the presence of SFE_com extract, there are 28 proteins that become upregulated compared to control, like MACF1 (microtubule-acting cross linking factor 1), which is 3-fold increased or ~2-fold CDH1 (Cadherin1) and 14 that get downregulated in the secretome (for example TALDO1 transaldolase, 3-fold down). For the SLE_com treated cells, the secretome reveals 87 upregulated proteins, like FSTL1 (Follistatin related protein 1), which increases 5-fold and 58 downregulated like ENO1 (Alpha-Enolase enzyme), which, instead, decreases 5-fold. Lastly, for the SLE_cult treated cells, 62 proteins were detected to be upregulated in a statistically significant way, like EMILIN1 adhesion protein 3.6-fold (also detected in the SLE_com dataset), and 39 targets were downregulated, including FABP3 at -3.4-fold (Fatty acid-binding protein 3), as shown in Supplementary Table S1.

**Figure 5:**
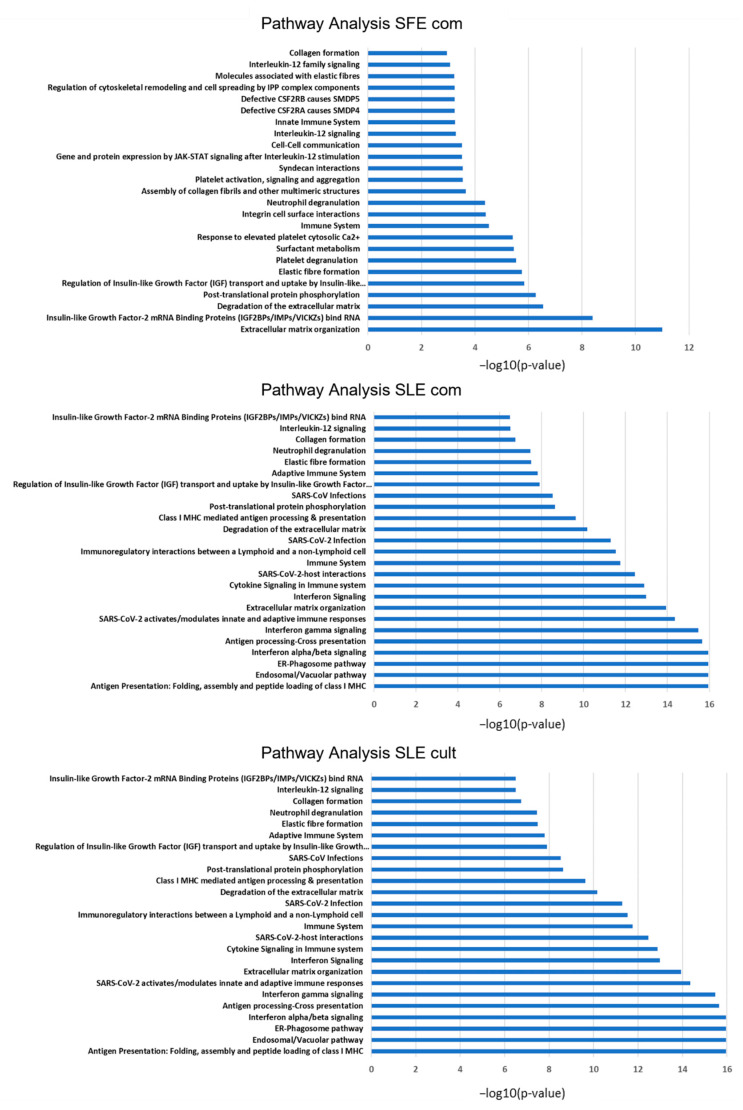
Bar graphs showing the pathway analysis that was performed using the Reactome pathway database in TNFα-activated MSCs treated with the *C. vulgaris* extracts. Secretome from SFE com-treated MSCs is depicted in the top panel, mid panel is for the SLE com, and bottom panel for the SLE cult samples. The length of the bars is relative to the statistical significance (−log of the *p* value).

Pathway analysis using all differentially regulated proteins from each dataset (both up and downregulated subsets) in the online tool Reactome (https://reactome.org/, Version 83 released on 7 December 2022) provided clues about the affected pathways when MSCs are pre-treated with extracts before being exposed to inflammatory stimulus. Interestingly, this analysis showed that generally *C. vulgaris* extracts affected pathways related to inflammatory response in TNFα- activated MSCs, as well as immunomodulatory pathways (signaling to Neutrophils) and extracellular matrix organization (Figure 5). Collagen formation was one of the pathways upregulated with all three extracts in the licensed MSCs, and the same holds true for Interleukin-12 signaling, which is crucial for the crosstalk between cells of the immune system. The endosomal/vacuolar pathway and antigen presentation are prominently represented in both SLE_com and SLE_cult datasets, but not the SFE_com, which has a milder effect (fewer differentially regulated hits) and mainly affects extracellular matrix organization (Figure 5). This cumulative dataset provides the basis and future reference for further follow-up mechanistic studies on the effect of the algal extracts on human adipose-derived MSCs (see Discussion). 

## 3. Discussion

MSCs hold great therapeutic potential, but for a variety of reasons, it has yet to be fulfilled. Among the reasons could be the effect of oxidative stress on MSCs’ ex vivo proliferation. This could lead to problems with function and engraftment in vivo. Oxidative stress is characterized by deregulated production and/or scavenging of reactive oxygen and nitrogen species (ROS and RNS, respectively). Antioxidants counteract the harmful effects of ROS and consequently treat oxidative stress-related diseases. Few antioxidants including edaravone, nacetylcysteine, alfa-lipoic acid and some flavonoids and oxerutins (for chronic venous insufficiency), as well as baicalein and catechins are being used clinically. Recently, nitrones had been used, but have not passed the scrutiny of clinical trials. 

The above fact had led to alternative efforts of identifying new therapeutic approaches by using natural-derived compounds [28]. Comparisons of microalgae with other sources of antioxidant bioactive compounds such as bacteria, yeast, insects, plants and human cell lines demonstrate that microalgae are an inexpensive source of bioactive compounds, non-susceptible to human pathogens, with low risk of contamination and high efficiency. Moreover, glycosylation of the bioactive compounds is low, while the production time of the high valuable antioxidants is ranging from weeks to months. Harvesting and purification of these antioxidant compounds, as well as the genetic manipulation of the microalgae species and the scale-up technology, need to be improved. Despite the fact that the role of microalgae to the antioxidant treatment market is less than 10%, there is a high possibility for further improvement and usage of microalgae extracts as antioxidant agents [29].

The biomass profile of the commercial and cultivated *C. vulgaris* verified in our study that protein content prevailed over carbohydrates and lipids in accordance with the literature [21,22,23,24,25,26,27,28,29,30,30,31,32,33,34,35], taking into account possible differentiation in cultivation and harvest conditions. Ash content of both commercial and cultivated biomass was consistent with literature [33,34,35], while low moisture content allowed long-term safe storage. The primary composition of the biomass supports the antioxidant activity of the obtained microalgae extracts. Moreover, the enhanced chlorophyll content of the SLE com. extraction method is possibly due to the polar affinity of ethanol 90% *v*/*v* with chlorophylls b and c [36,37].

In MSCs, excess ROS or exogenous addition of H_2_O_2_, which is a widely used procedure to cause oxidative damage/stress in cellular models [38], can impair self-renewal, differentiation capacity, and proliferation [39], as this is also identified in our analysis at 300 μM of H_2_O_2_, which reduces cell viability; concordantly, antioxidants stimulate MSC proliferation [40]. MSCs have multimodal roles related with immunomodulatory functions which suppress proliferation of T cells and NK cells and dampen inflammation progression [41]. Moreover, they have been used extensively in tissue generation, damage healing, as well as improving engraftment of other cells and tissues. These roles of MSCs are being compromised upon ROS generation, leading to MSCs damage and dysfunction. This primarily is the reason for trying to understand the contribution of the oxidative stress to MSCs biology and biochemistry and to try to define solutions [42].

*C. vulgaris* extracts can protect MSCs from H_2_O_2_-induced oxidative stress either by reducing the production of ROS with their free radical scavenging effect or by improving the antioxidant capacity of the cells as identified in our study. The different extraction methods of the microalgae resulted in enhanced antioxidant activity of the supercritical extract followed by the solid-liquid extract from the commercially available *C. vulgaris* and then by the SLE extract from the laboratory cultivated *C. vulgaris*. According to the extracts characterization in terms of several bioactive compounds, ROS reduction for each one of the three different extraction methods is possibly related with the total phenolic levels. The sufficient ability of the laboratory cultivated *C. vulgaris* extracts in attenuating ROS production supports an investment in future efforts for fast, economical and efficient treatments of oxidative stress in human cell cultures with the potential to impact the potency of emerging cell therapies. 

Acetone extracts from *C. vulgaris* in other studies have resulted in antioxidant activity via DPPH assay measurement at almost 51% [19], while the 90% ethanol extracts in our analysis resulted in a similar or even better antioxidant effect, while the SFE extract demonstrated an even robust phenotype. Aqueous extracts from various microalgae species showed reduced antioxidant activity [20] compared to the ethanol and supercritical extracts due to reduced recovered phenolic compounds. Culture conditions, light and even the identity (phenolic acids, flavonoids, phlorotannin, halogenated phenolic compounds) of each specific compound and more specifically the phenolic content may affect the production rate of the antioxidant bio-active compounds [43]. The absence of any cytotoxicity of the microalgae extracts renders them an important source for targeting the oxidative phenotype without any side effects to the basal cellular functions of the MSCs. Our study is a preliminary functional in vitro effort to prove the antioxidant capacity of *C. vulgaris* extracts on MSCs oxidative stress offering novel ways to optimize ROS levels, which is urgently needed in order to fully exploit immunomodulatory and regenerative capabilities of these cells. However, more detailed studies employing-omics technologies such as proteomics are required to reveal the extracts’ mechanism of action and the mitochondrial pathways involved against oxidative stress. Taken together, our results underline the high added value of *C. vulgaris* extracts that could offer a wide range of new applications in the biotechnology sector.

Our exploratory proteomics dataset revealed that pre-incubation of mesenchymal stromal cells with the extracts (especially the SLE com and SLE cult rather than the SFE com) leads to a discernible differential signature in the secretome of the cells upon inflammatory stimulation, with upregulation of the anti-inflammatory, immunomodulatory and extracellular remodeling pathway. It is noteworthy that many membrane-associated cell adhesion proteins are detected as being upregulated (e.g., Cadherin, EMILIN1, Integrins), which, when taking into consideration that this comes from the acellular secretome of MSCs rather than cells themselves, indicates that vesicle secretion (exosomes etc.) increases in the presence of algal extracts, which may be beneficial for the complex cell–cell communications. Strengthening this notion, the Reactome pathway analysis clearly depicts the endosomal/vacuolar pathway as a significantly regulated group, and exosomes are known to be generated from this pathway [44]. The resource provided with this paper (Supplementary Table S1) can form the basis for future mechanistic investigations and help to shed light on the functional consequences for the cells that receive the extracts. It can be envisioned that, apart from developing these algal extracts for use in the supplement or cosmetic industry, they could be of interest in the process development context of an advanced cell therapy with adipose-derived MSCs. For instance, it could be investigated whether the pre-incubation of the cells with the extracts described here (or a defined subset) has the potential to enhance the Critical Quality Attributes (CQAs) of the MSC product, such as superior performance in potency assays, which might eventually translate to an ameliorated therapeutic effect in clinical trials. This would be very impactful, taking into consideration that MSCs are being developed as therapy for a variety of human (and animal) maladies. 

## 4. Materials and Methods

### 4.1. Materials/Chemicals

In this study, commercially available *C. vulgaris* biomass as well as biomass from *C. vulgaris* cultivated in the lab was utilized. The commercially available *C. vulgaris* biomass was purchased from Go Superfoods Ltd. (Sheffield, UK). Specifically, commercial *C. vulgaris* was cultivated in natural water open ponds in South China, collected with mesh screens, and subjected to milling and spray drying according to the regulations for human consumption-intended products. *C. vulgaris* strain (UTEX 1809) was purchased from Culture Collection of Algae at the University of Texas (Austin, TX, USA).

Carbon dioxide (99.5%) was purchased from Air-Liquide Hellas (Athens, Greece). Ethyl acetate, orthophosphoric acid (analytical grade reagents), methanol (≥99.8%), tert-butyl-methyl ether (MTBE), water (HPLC grade reagents), and anhydrous sodium carbonate (99.5%) were purchased from Fisher Scientific International Inc. (Pittsburgh, PA, USA). The standard compounds of astaxanthin (≥98%), lutein (≥92%), and β-carotene (≥95%) for HPLC analysis were purchased from Acros Organics BVBA (Antwerp, Belgium), Extrasynthese SAS (Lyon, France), and Sigma Aldrich Co. (Saint Louis, MO, USA) respectively. Gallic acid (98%) and anhydrous D(+)-glycose (≥99.8%) (ACS reagents) were purchased from Acros Organics BVBA (Antwerp, Belgium) while analytical grade potassium chloride was purchased from Panreac Quemica SA (Barcelona, Spain). Finally, the free radical 2,2-diphenyl-1-picrylhydrazyl (DPPH) and Folin–Ciocalteu reagent were purchased from Sigma Aldrich Co. (Saint Louis, MO, USA) and Carlo Erba Reagents SAS (Milan, Italy), respectively. DMEM-F12 medium, FBS and PBS were purchased by Fisher Scientific International Inc. (Pittsburgh, PA, USA). DCF-DA (2′,7′-dichlorofluorescin diacetate) was purchased by Sigma Aldrich Co. (Saint Louis, MO, USA). 

### 4.2. Instrumentation

The instrumentation used during this study is listed below. A Gallenkamp OVA031.XX1.5 vacuum oven (A. Gallenkamp & Co., Ltd., England, UK) is used for drying processes, while a Thermolyne 47,900 furnace (Barnstead Thermolyne Corp., Osseo, MN, USA) is used for combustion. Ultrasound application is performed at 35 kHz and ambient temperature in an Elma D-7700 Transsonic Digital ultrasonic bath (Elma Schmidbauer GmbH, Singen, Germany). An Eppendorf 5452 Mini Spin centrifuge (Eppendorf AG, Hamburg, Germany) is used for small volume sample centrifugation, while a Hermle centrifuge Z206-A (Hermle AG, Ba-den-Württemberg, Germany) is used for larger volume samples. ChromPure PTFE/L 0.45 μm filters (Membrane solutions, LLC., North Blend, OH, USA) are used for all required filtrations. A Carousel tech stirring hotplate (Radleys, Essex, UK) is used for stirring purposes while a Vortex-Genie^®^ 2 mixer (Scientific industries Inc., Bohemia, NY, USA) is used for intense stirring. A Hei-VAP Advantage ML rotary evaporator (Heidolph Instruments GmbH & Co. KG, Bayern, Germany) is used for solvent evaporation. All the required absorbance measures for the determination of biomass’ lipids and proteins as well as extract’s total phenolic, chlorophyll, carotenoid content and antioxidant activity are executed in a Shimadzu UV-1900i UV-Vis Spectrophotometer (Shimadzu Corporation, Kyoto, Japan) using 1 cm length quartz cuvettes. An apparatus comprising of a Speed Digester K-425, a Scrubber K-415 for exhaust gas collection and a Kjelflex K-360 distillation device (Buchi Labortechnik AG, Flawil, Switzerland) are used for nitrogen determination. Identification and quantification of carotenoids are performed through high performance liquid chromatography in an HPLC device that includes a Jasco LG-1580-04 gradient unit and a Jasco PU-1580 HPLC pump (Jasco Inc., Easton, MD, USA), a Rheodyne 7125 injector (Rheodyne Europe GmbH, Bensheim, Germany) with 20 μL loop, a Jones 7955 column chromatography heater (Jones Chromatography Limited, Wales, UK) and a Shimadzu SDP-M20A Diode Array Detector (DAD) (Shimadzu Corporation, Kyoto, Japan). The stationary phase is immobilized in a YMC C30 reversed-phase column, 5 μm, 250 × 4.6 mm I.D., (YMC Co., Ltd., Kyoto, Japan). Finally, supercritical CO_2_ extraction is carried out in a SFE-500 bench scale apparatus (SEPAREX CHIMIE FINE, Champigneulles, France), details of which are described in previously published work [45]. The absorbance at 450 nm is determined by a multiplate reader (Infinite F50, TECAN). The fluorescence of DCF is measured using Arthur™ Image Based Cell Analyzer (NanoEnTek Inc., Seoul, South Korea).

### 4.3. Methods

#### 4.3.1. Cell Culture and Reagents

The cultivated strain of *C. vulgaris* is grown in a medium as previously described by Savvidou et al. [9]. The pH, the temperature and the light intensity are set to 7.0, 24 °C and 50 mol photon m^−2^ s^−1^, respectively. Human Mesenchymal Stem Cells from Adipose Tissue (hMSC-AT) are obtained from PromoCell (Prod. Nr. C-12977) and are cultured at 37 °C and 5% carbon dioxide in DMEM-F12 medium supplemented with 10% FBS. The medium is replaced twice per week, and the cells are split enzymatically with Trypsin-EDTA (0.05%) (Gibco Life Technologies). Cultures were trypsinized when the cells were approximately at 80–90% confluency.

#### 4.3.2. Biomass Characterization

Biomass characterization included moisture, ash, lipid, carbohydrate, and protein content determination. All the assays performed for the biomass characterization are described in detail in previous work [25]. In summary, moisture and ash content are determined through vacuum drying and combustion, respectively, until weight stabilization. The Folch protocol is performed for lipid content determination [46], according to Araujo et al. [30], with additional scale-down adjustments. The phenol-sulfuric method is applied according to Moheimani et al. [47] for the carbohydrate content determination. Finally, protein content is determined through the Kjeldahl method [48], as adapted by Büchi Labortechnik AG (Flawil, Switzerland) [49]. All measurements are performed in triplicate, and results are accompanied by their standard deviation. Moisture content is expressed as a dry-to-wet mass percentage (% *w*/*w*), and the rest of the values are determined on a dry basis (% dw).

#### 4.3.3. Biomass Extraction

##### Supercritical Fluid Extraction (SFE) 

The supercritical fluid extraction procedure using CO_2_ as solvent followed the detailed description from Papamichail et al. [45]. Approximately 80 g of commercially available *C. vulgaris* biomass are loaded in the extractor vessel. Glass beads (d = 4.5 mm) are added above and below the biomass and contributed to the reduction of the extractor dead space and the accomplishment of uniform flow distribution. The extractor operates at 250 bar, 60 °C and with a solvent flow rate set at 40 g/min, while the two separators are set to 60 and 10 bar, respectively and 8 °C. Moreover, total solvent consumption is set to 100 kg_CO2_/kg_biom_ for maximum extract recovery. The proposed extraction conditions are defined following an optimization process, as presented by Georgiopoulou et al. [25] (Table A1—Appendix B). Extraction yield is determined gravimetrically through weight loss of biomass. The obtained extracts are stored at −18 °C for further analysis. 

##### Solid-Liquid Extraction (SLE) 

The conventional extraction procedure is applied to both commercially available and laboratory cultivated biomass. Approximately 1 g of *C. vulgaris* biomass and 37 mL of aq. ethanol, 90% *v*/*v* (ratio: 37 mL/g) are loaded into a jacketed vessel, heated at 30 °C and stirred at 500 rpm for 24 h in the dark. The proposed extraction conditions have been defined following an optimization process, as presented by Georgiopoulou et al. [25] (Table A1—Appendix B). Extraction yield is determined gravimetrically through the extract’s mass. The obtained extracts, after vacuum evaporation, are temporarily refrigerated at −18 °C prior to analysis.

#### 4.3.4. Extract Characterization

All the algal extracts are subjected to determination of their antioxidant activity, phenolic, chlorophyll and carotenoid content, as well as concentration in selected carotenoids of great interest, i.e., lutein, astaxanthin, lutein and β-carotene. All the assays performed for the extract characterization are described in detail in a previous publication [25]. In summary, the DPPH free radical scavenging method is performed according to Laina et al. [50] for antioxidant activity determination. The half-maximal inhibitory concentration (IC_50_) is detected at 515 nm and expressed in mass ratio of extract to DPPH (mg_extr_/mg_DPPH_). 

Determination of total phenolic content (TPC) is performed through the Folin–Ciocalteu method, according to Drosou et al. [51]. Detection is performed at 765 nm and phenolic compounds are expressed as gallic acid equivalent mass per extract mass (mg_GA_/g_extr_). Total carotenoid and chlorophyll content is determined through correspondingly appropriate equations provided by Jeffrey et al. [52,53] (see Appendix C). Carotenoid and chlorophyll quantification require absorbance measurements at 480, 510, 630, 647 and 664 nm, and final content expression is in mass ratios of the corresponding compound to extract (mg/g_extr_). 

The received extracts are also subjected to RP-HPLC for carotenoid determination of the selected carotenoids of astaxanthin, lutein and β-carotene. Retention time and absorbance spectra of external standards (Appendix A) are used for identification of the selected carotenoids, while quantification emerged from corresponding standard reference curves. Methanol, methyl tert-butyl ether (MTBE) and aq. phosphoric acid, 1% *v*/*v*, consisted of the mobile phase according to a linear gradient reported by Stramarkou et al. [54]. The concentration of aq. phosphoric acid, 1% *v*/*v*, is set at 4%, while the linear gradient for methanol is set as follows: 0 min, 81%; 15 min, 66%; 23 min, 16%; 27 min, 16%; 27.1 min, 81%; 35 min, 81%. During analysis, the column temperature is set at 35 °C and mobile’s phase flow rate at 1 mL/min. Finally, concentrations of astaxanthin, lutein and β-carotene are summed, and selected carotenoid content is expressed in a mass ratio of compound to extract (mg/g_extr_).

#### 4.3.5. Solubility of *C. vulgaris* Extracts Obtained by Different Extraction Methods

**Figure 6:**
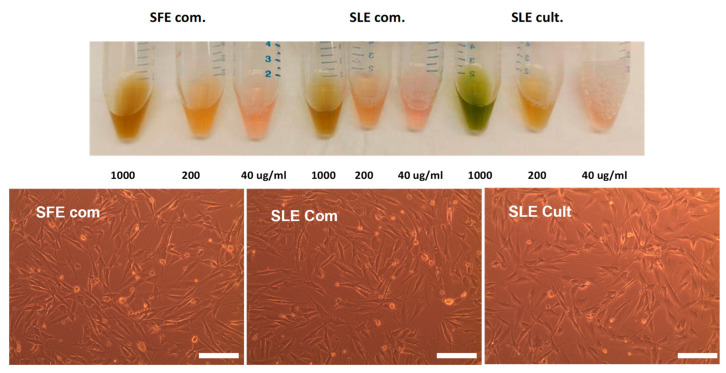
Solubility of *C. vulgaris* extracts obtained by different extraction methods. **Upper panel:** Representative pictures of serial dilutions of *C. vulgaris* extracts in DMEM/F12 10% FBS medium. **Bottom panels**: Microscope images of MSCs incubated with extracts SFE com, SLE com, SLE cult, showing that the extracts are well soluble in medium at 50 μg/mL, as no insoluble aggregates are observed. 10X objective, Leica DM IL. Scale bar, 100 μm.

The constituents and the antioxidant activity of the extracts verified previously via the DPPH free radical scavenging assay prompted us to proceed with testing them in a cell-based assay. It is necessary to identify the best dilution of the three different microalgae extracts in cell culture medium in order to perform in vitro experiments for testing the antioxidant properties of the different extracts. Initially, the extracts are dissolved in 100% ethanol with a concentration of 20 mg/mL. At this concentration, we achieve good solubility, while higher concentrations are unsuccessful due to the fact that a significant part of the mass remained insoluble. From the 20 mg/mL stocks, successive dilutions in DMEM-F12 10% FBS medium: 1000 μg/mL (5% ethanol), 200 μg/mL (1% ethanol) and 40 μg/mL 0.2% ethanol are generated (Figure 6). Since ethanol is known to be toxic for cultured cells when used above 1%, final concentration in the medium, but completely tolerable below 1%, for the in vitro cell-based experiments concentrations below 200 μg/mL are used in order to minimize ethanol-derived toxicity. 

#### 4.3.6. Cell Viability Assay

Cell viability is measured by Cell Counting Kit-8 (CCK-8, Sigma-Aldrich, Munich, Germany). Cells are seeded into 96-well at density 3 × 10^4^ cells/cm^2^ cultured in DMEM-F12 medium with 10% FBS (3 biological replicates in each condition). After 24 h, cells are treated with the microalgae extracts in three different concentrations (150 μg/mL, 50 μg/mL and 16.7 μg/mL) related with good solubilization as previously identified. After 24 h, cells are washed, and fresh DMEM-F12 medium with 10% FBS is added for an additional 24 h. CCK-8 solution (10 μL) is added to each well, followed by incubation for 2 h at 37 °C. The absorbance at 450 nm is determined by a multiplate reader (Infinite F50, TECAN). Cell viability is expressed as a normalized percentage relative to the control (untreated) cells.

#### 4.3.7. Measurement of Intracellular Reactive Oxygen Species

Cells are seeded into 24-well at density 3 × 10^4^ cells/cm^2^ and cultured in DMEM-F12 medium supplemented with 10% FBS. Cells are treated with the microalgae extracts (50 μg/mL) for 24 h and stimulated with 300 μM hydrogen peroxide for 2 h, followed by 2 h recovery in fresh medium. ROS-sensitive DCF-DA (2′,7′-dichlorofluorescin diacetate; Sigma Aldrich; 10 μM) is added for 20 min. Serum-free medium is used to perform 2 washes of the cells before trypsinizing and resuspending in 100 μL PBS. For quantification of ROS generation, the fluorescence of DCF is measured using Arthur™ Image Based Cell Analyzer (NanoEn Tek). Fluorescence signal is calibrated using the unstained sample as the autofluorescence threshold. The threshold is kept steady through all measurements. The filters used for fluorescence measurement are Ex 466/40 and Em 525/50. 

#### 4.3.8. Proteomics Analysis

For detecting differentially regulated secreted proteins from mesenchymal stromal cells treated with the algal extracts, the cells were seeded in T25 flasks at 2000 cells/cm^2^ density and treated with the different extracts at 50 μg/mL (in a total of 5 mL of medium) for 48 h. As reference control, a flask was left untreated (vehicle control, 1% EtOH). Then, the medium with the extracts was washed away and replaced with 5 mL of serum-free medium containing 10 ng/mL TNFα as inflammatory stimulus (licensed MSCs). The 4 cultures (3 with extract pre-incubation and 1 as control without pre-incubation, all with TNFα stimulation) were left for another 48 h in the serum-free medium to secrete proteins and extracellular vesicles. The conditioned medium (also referred to as “secretome” was collected, centrifuged at 300× *g* for 5 min to remove large debris and filtered using a 20 μm filter. SDS was added at 0.1% as a mild lysis agent to permeabilize vesicles. The secretome samples were snap-frozen at −80 °C and delivered to the Proteomics facility of the Biomedical Research Center Alexander Fleming in Vari, Athens for analysis, (as an outsourced paid service) using an LC-MS/MS Orbitrap method and Data-Independent Acquisition (DIA method) as previously described [55]. The data were analyzed using the Perseus 1.6.15.0 software from 2 independent replicate experiments. 

#### 4.3.9. Statistical Analysis

All experiments are replicated at least three times and data from parallel cultures are acquired. Statistical significance of groups is calculated by a one-way ANOVA test followed by Tukey or Dunnett’s post hoc test using GraphPad Prism 9 software. *p*-values < 0.05 were considered significant; * *p* < 0.05, ** *p* < 0.01, *** *p* < 0.001, **** *p* < 0.0001.

## 5. Conclusions

Microalgae derived antioxidant bioactive extracts demonstrated an inexpensive, non-susceptibility to human pathogens, with a low risk of contamination and a high efficiency approach compared to other sources. Antioxidants counteract the consequences of reactive oxygen species (ROS) and attenuate oxidative stress. In this study, 300 μM of H_2_O_2_ reduced cell viability of mesenchymal stem cells verifying an H_2_O_2_ effect on oxidative stress of mitochondria. Three different extracts of *C. vulgaris,* commercially available and laboratory cultivated in the lab, led to reduced levels of ROS in mesenchymal cells, without affecting their proliferation. Specifically, two different types of biomass are employed, i.e., commercially available *C. vulgaris* and laboratory cultivated in the lab *C. vulgaris* biomass. The first one is subjected to solid-liquid extraction with aq. ethanol and supercritical fluid extraction with CO_2_, while the latter is treated with aq. ethanol. The supercritical extraction proved to be the most efficient extraction method for carotenoids. In conclusion, pre-incubation of MSCs with *C. vulgaris* extracts strengthened their defense against the subsequent oxidative attack by H_2_O_2_ and may constitute a promising research avenue to enhance the potency of these cells which are clinically relevant as cell therapy for various human ailments. Our findings also suggest that *C. vulgaris* extracts may enhance the anti-inflammatory activity of mesenchymal stromal cells. The mechanisms of action responsible for the anti-inflammatory activities of these extracts are currently unknown; additional research is required to isolate and identify some active compounds that may be responsible for the activity, as well as to investigate the mechanism of action.

## Figures and Tables

**Table 1:** Primary composition of the commercially available and laboratory cultivated *C. vulgaris* biomass [25].

Primary Composition (% ^1^)	Commercially Available [25]	Laboratory Cultivated
Lipid	22.17 ± 0.46	17.64 ± 0.13
Carbohydrate	33.84 ± 1.33	19.51 ± 0.90
Protein	44.48 ± 0.77	39.70 ± 0.18
Ash	5.63 ± 0.06	10.70 ± 0.01
Moisture	2.32 ± 0.12	1.15 ± 0.09

^1^ All values except moisture are expressed on dry basis (dw). Moisture is expressed as percentage of a dry-to-wet mass percentage (% *w*/*w*). Data represent mean ± SD (standard deviation, *n* = 3 independent experiments).

**Table 2:** Extract characterization of SLE and SFE derived from *C. vulgaris* biomass.

Response	SLE cult.	SLE com. [25]	SFE com. [26]
Yield (% *w*/*w*)	17.53 ± 0.54	15.39 ± 0.54	3.37 ± 0.07
Total phenolic content (mg_GA_/g_extr_)	14.88 ± 3.61	18.23 ± 3.61	18.29 ± 2.05
Total chlorophyll content (mg/g_extr_)	87.27 ± 2.48	53.47 ± 2.48	32.55 ± 1.54
Chlorophyll a (mg/g_extr_)	32.49 ± 1.77	36.61 ± 1.77	32.55 ± 1.54
Chlorophyll b (mg/g_extr_)	44.45 ± 1.02	13.92 ± 1.02	-
Chlorophyll c (mg/g_extr_)	10.33 ± 0.32	2.94 ± 0.32	-
Selected carotenoid content (mg/g_extr_)	15.37 ± 0.13	4.12 ± 0.13	10.00 ± 0.30
Astaxanthin (mg/g_extr_)	0.503 ± 0.020	0.430 ± 0.020	0.155 ± 0.022
Lutein (mg/g_extr_)	13.92 ± 0.13	3.40 ± 0.13	8.78 ± 0.32
β-carotene (mg/g_extr_)	0.952 ± 0.011	0.290 ± 0.011	1.07 ± 0.24
Total carotenoid content (mg/g_extr_)	27.03 ± 0.52	9.92 ± 0.52	21.14 ± 1.39
Antioxidant activity—IC_50_ (mg_extr_/mg_DPPH_)	69.72 ± 5.52	52.58 ± 5.52	44.35 ± 4.32

Data represent mean ± SD (standard deviation, *n* = 3 independent experiments).

**Table 3:** Retention time of the selected carotenoids of the examined extracts analyzed by RP-HPLC.

		Identified Carotenoids		
		Astaxanthin	Lutein	β-Carotene
SLE cult.	Retention time (min)	6.3	9.9	23.9
SLE com.		7.1	9.6	24.0
SFE com.		7.0	10.0	24.2

**Figure A1:**
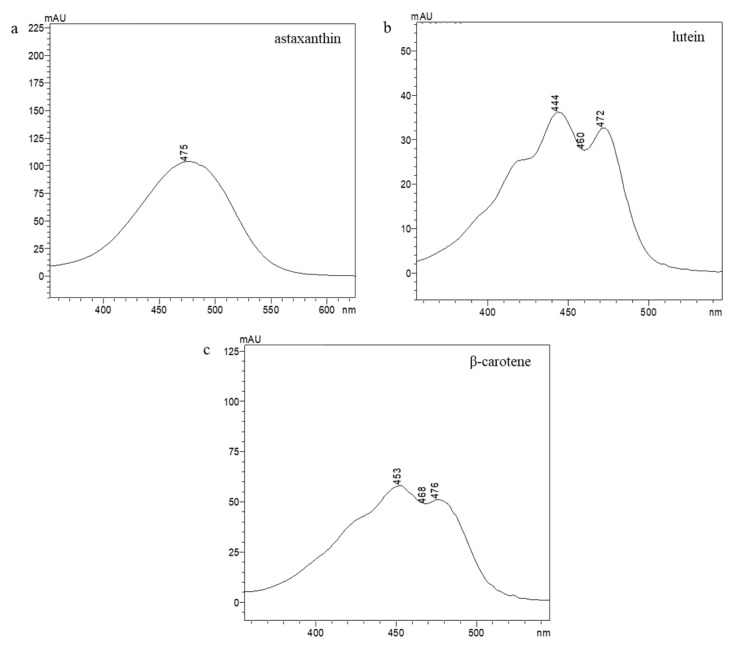
Absorbance spectra of (**a**) astaxanthin; (**b**) lutein and (**c**) β-carotene.

## Data Availability

The data is contained within the manuscript and a Supplementary Table S1 is provided online.

## Supplementary Materials

The following supporting information can be downloaded at: https://www.mdpi.com/article/10.3390/plants12020361/s1, Table S1: Table of protein hits detected by the proteomic analysis of secretomes from stimulated MSCs pre-treated with microalgal extracts.

## Appendix A. Identification of the Selected Carotenoids through RP-HPLC

The distinctive absorbance spectra of external standards of astaxanthin, lutein and β-carotene presented in Figure A1 contributed to the identification of the selected carotenoids.

## Appendix B. Operational Conditions of SLE and SFE

The operational conditions of the SLE and SFE of bioactive compounds from *C. vulgaris* are gathered and presented in Table A1.

**Table A1:** Optimum operational conditions of SLE and SFE.

Parameter	SLE cult. SLE com. [25]	SFE com. [26]
Solvent	Ethanol 90% *v*/*v*	CO_2_
Biomass	Laboratory cultivated/ Commercially available	Commercially available
Solvent-to-biomass (kg/kg_biom_)	30	100
Stirring (rpm)	500	-
Duration (h)	24	3.3
Temperature (°C)	30	60
Pressure (bar)	1	250
Solvent flow rate (g/min)	-	40

## Appendix C. Supplementary Data of Total Chlorophyll and Carotenoid Determination

Determination of chlorophyll and carotenoid content was performed through the equations provided by Jeffrey and Humphrey [52] as presented below:

c_a_ = 11.85 × Abs_664_ − 1.54 × Abs_647_ − 0.08 × Abs_630_ (A1)

c_b_ = 21.03 × Abs_647_ − 5.43 × Abs_664_ − 2.66 × Abs_630_ (A2)

c_c_ = 24.52 × Abs_630_ − 1.67 × Abs_664_ − 7.60 × Abs_647_ (A3)

c_chlor._ = c_a_ + c_b_ + c_c_ (A4)

c_carot._ = 7.60 × Abs_480_ − 1.49 × Abs_510_ (A5)

where c_a_, c_b_, c_c_, c_chlor._ and c_carot._ stand for the concentration of chlorophyll a, b, c, total chlorophylls and total carotenoids, respectively (μg/mL).

Mass units (g_extr_/mL) were used for the expression of total chlorophyll (C_chlor._) and carotenoid (C_carot._) as presented below:

C_i_ = 10^3^ × c_i_/C_sample_, i = chlor., carot. (A6)

where C_sample_ stands for the sample concentration (g_extr_/mL).
